# A Winter Cold

**Published:** 1882-10

**Authors:** F. H. Bosworth

**Affiliations:** N. Y. City; Professor of Diseases of the Throat, in the Bellevue Hospital Medical College, New York


*FOR CONTENTS OF THIS NUMBER SEE PAGE* 592.

*THE*

### Independent Practitioner.

VOL. III.——*October*, 1882.——NO. 10.

### ORIGINAL COMMUNICATIONS.

*We are not responsible for the opinions expressed by contributors.*

ARTICLE I.

### A WINTER COLD.

BY F. H. BOSWORTH, M. D., N. Y. CITY.

Professor of Diseases of the Throat, in the Bellevue Hospital Medical College, New York.

During the Fall and Winter months we are all of us called upon frequently to advise and prescribe for patients who, as a result of an exposure to cold, have contracted what is ordinarily called a bronchitis. If the constitutional symptoms are of a prominent character the affection is oftentimes spoken of as "threatened with pneumonia." In the milder cases it is simply a "bad cold." The ordinary procedure in these cases is to make a careful chest examination by means of percussion and auscultation, with the result of finding nothing abnormal, and which, therefore, teaches us nothing more than that there is serious disorder of the lungs, and so we pronounce it a case of bronchitis, write a prescription for a cough mixture and let our patient get well in the natural course of events. If, however, we make a more thorough examination we are enabled to discover the exact extent and limit of the morbid process, and as a rule will find that the mucous membrane lining the upper air passages from the anterior nares to the trachea are in a state of active acute inflammation. The mucous lining of the nasal cavity will be seen by anterior inspection and posterior rhinoscopy swollen and turgid, the surface coated by greyish mucus or mucopus according to the duration of the attack, and the nasal cavity markedly encroached upon, and the normal nasal respiration interfered with or arrested by the narrowing of the passage. The pharynx, palate, uvula and faucial pillars will also present an angry, red and swollen appearance, their surface being covered with an active mucous discharge. The tonsils also in many cases will be moderately swollen and project from their bed. The larynx will be found to present evidences of acute inflammation, its lining membrane being reddened, slightly swollen and covered with ropy mucus, the vocal cords being of the same bright red color as the remainder of the mucous lining. If a view is obtained below the open glottis we will find the tracheal lining of the same bright color, the yellow color of the cartilaginous rings which is ordinarily seen in health, being completely obscured by the congested mucous membrane, while the normal depressions between the rings are to an extent obliterated. If we were to give an anatomico-pathological name to the above condition we might call it an acute catarrhal naso-pharyngo-laryngo-tracheitis. I prefer to designate it as a winter cold, as more expressive of the clinical aspect of the affection.

The onset of a winter cold is generally marked by a feeling of chilliness rather than a well marked chill. This is soon followed by a general febrile movement, characterized by a hot skin, rapid pulse, flushed face, headache and nausea, the temperature of the body often attaining 102° 103°. In connection with this there is a sense of oppression referable to the sternal region, and some slight difficulty in breathing. The nasal cavities become occluded, there is a swollen and painful feeling in the fauces, with discomfort rather than pain in swallowing. The voice is either husky or hoarse. There is also a cough which, at the onset of the attack, is peculiarly distressing on account of its dry and wheezing character, and from the fact that it accomplishes nothing in the way of clearing or relieving the oppressed air passages. As the affection progresses, however, and secretion becomes freer, the cough becomes looser, as it is called, and less distressing. The secretion, at first scanty, after one or two days becomes free, and consists of a gray glairy mucus. Still later it becomes muco-purulent. The affection runs its course in a week or ten days, unless retarded either by indiscretion or a fresh cold, and undergoes resolution, or it lapses into a more or less obstinate and chronic cough.

The direct cause of a winter cough is taking cold. We ordinarily regard a simple cold as a somewhat trival affair, and yet if we ask ourselves what it is, and how it is contracted, we will find the question an exceedingly difficult one to answer.

Many theories have been advanced in explanation of the phenomena of a cold, among others that of Rosenthal may be noticed.

His theory is that the immediate effect of cold, acting on the surface of the body, is to excite contraction in the peripheral vessels, by which the blood is driven from the surface in upon the internal organs, and acts there as an irritant, exciting inflammation. This view of the matter is somewhat mechanical, and scarcely explains the action of cold in many instances. Not unfrequently as the result of an exposure, it is not really internal organs that become the seat of the resultant inflammation, as an attack of acute eczema, or acute conjunctivitis may result. Or, in the case of the commonest of all inflammatory affections resulting from exposure, an attack of acute coryza affects a membrane so near the surface that under the action of Rosenthal's theory, the blood should, to an extent at least, be driven from the membrane rather than that it should be flushed upon it from without. Furthermore, as we know, mere mechanical congestion does not lead to true inflammatory action, as shown by the old familiar and often repeated experiment of ligating the efferent veins of the frog's foot, and observing the result in the web under the microscope.

A far more plausible view of the matter is that of Seitz. His theory is that disorders resulting from catching cold are due to the removal of heat to an unusual extent from the external or internal surface of the body, that this causes some functional disturbance which, in its turn, gives rise to certain morbid processes distant from the part immediately affected by the cold. That the morbid changes are not due to the immediate or direct effect of this exposure is evident from the fact that as a rule a certain length of time elapses before these changes set in.

The theory of Seitz, it seems to me, is not complete, but leaves the matter somewhat in the dark. The true action of cold upon the body in producing morbid conditions, is probably in those nutritive changes which are constantly going on, and by which the animal heat is developed. This heat production is going on in all the tissues of the body. In order that this function shall not be impaired, it is necessary that the normal temperature shall be maintained. This we know is 98⅝°. Any marked deviation from this normal standard as the result of extraneous influences, results in morbid changes. If heat production is arrested in a portion of the body under the action of an intense cold, molecular death of the part ensues, as is the case when gangrene of a limb results from freezing. If the action of the cold is insufficient to arrest the nutritive processes of the part, it may cause only inflammatory action. In these cases we have only the direct action of a low temperature on the organism. In the ordinary phenomena of "taking cold," we have still the result of a low temperature acting on the heat producing processes, but in an indirect manner. The direct action of the cold is, as a rule, upon the surface of the body, but the resultant morbid condition is upon some organ remote from the exposed part. In both cases, however, the cause and effect are the same; and the connection between the exposure and resultant inflammatory condition is the disturbance of those nutritive changes in the tissues, which result n the production of animal heat.

There are three factors generally necessary for the production of "a cold;" low temperature, air in motion, and moisture. It is also necessary as a rule, that one or more of these factors should act for a somewhat long duration of time, as we know the momentary action of an intense cold or draft or moist atmosphere, does not usually result in any morbid changes, but that it is only after a somewhat prolonged exposure of the body that the familiar phenomena of a cold ensue. In our ordinary life there are few of us but that are subject to slight, temporary exposure with impunity; as for instance, upon rising in the morning in a cold room, changing one's clothes, etc. On the other hand, the sitting in a draft for a prolonged period with even only a small portion of the body exposed, may lead to serious or grave morbid changes. Among the most familiar causes of taking cold may be enumerated, sittiug in a draft, wearing insufficient clothing, wearing thin-soled shoes, insufficiently protected feet, going from a warm room to a cold room, slight exposure while perspiring, etc. Wearing thin-soled shoes or insufficiently protected feet, is a very prolific source of trouble, as the loss of heat in this manner is far greater than is usually recognized. Especially is this the case if the soles of the shoes are damp, as in this case, of course, the radiation takes place much more rapidly.

Again, when the body is perspiring, the loss of heat is going on with considerable activity; hence we find that, in this condition, even a slight exposure is liable to result in far more serious disturbance than would occur from the same exposure were the body not in an overheated condition. There should, however, be borne in mind this difference, if the perspiration is the result of violent exercise, all the nutritive processes are stimulated to an abnormal activity, animal heat is being generated rapidly, and the perspiration necessarily sets in as a conservative measure, to prevent too great accumulation of heat in the system, but still as the direct consequence of the violent exercise. If now, in this condition the body is exposed to the influence of cold, and the perspiration suddenly checked, very serious consequences may ensue. If, however, on the other hand, a copious perspiration is brought on by artificial means, while the body is in a state of quiescence, as in the hot room of the Turkish bath, the heat source is from without, the heat producing forces of the system are not disturbed, and the cold plunge, while of course it suddenly checks the perspiration, does not, as a rule, give rise to any untoward consequences. Moreover, the exposure by the cold plunge is only temporary and of short duration, and by the subsequent manipulations, any serious loss of heat which may have resulted is speedily and completely restored.

A swimmer may remain in water at a temperature twenty or thirty degrees below that of the body, and that too for a somewhat prolonged period of time, but while in the water he is in a state of constant and often laborious activity, thereby setting in play those processes by which animal heat is generated. But even with this constant activity, if the bath becomes too prolonged, there comes a time when the body is unequal to the task of supplying sufficient animal heat to make up for the loss, and the bather succumbs to the direct influence of this tremendous drain upon the system. As was said before, the loss of animal heat does not directly produce these morbid changes, but creates or gives rise to certain functional disturbances with the nature of which we are not entirely acquainted, and these give rise, after a certain interval of time, to the morbid changes which we call taking cold. This interval may be short, lasting perhaps but a few hours, as is usually the case in slighter disorders; or it may be prolonged one or two days or even more; in this case, as a rule, the resultant disorders are of a more serious character. There is generally attendant upon taking cold, fever of a more or less marked character. That this fever is not symptomatic but an essential fever, is shown by the fact that it stands in no constant relation to the morbid changes which result; as in even slight disorders we may have the febrile motion more marked than the fever which accompanies the more aggravated forms of inflammatory trouble which may arise from the cold. Moreover, the fever generally sets in immediately after exposure, and when the later morbid changes appear, no increase of fever, as a rule, is detected. As regards the local disorders which result from an exposure to cold, we find them manifesting themselves in any part of the body. We may have acute coryza, pharyngitis, gastric catarrh, muscular rheumatism, cystitis, or in fact an attack of inflammation, involving any of the organs of the body as the result of a cold. Owing to their exposed condition, being the first to receive the current of inspired air with its impurities or whatever of irritating qualities it may possess, the upper air passages are perhaps more subject to inflammation than any other portion of the body, and once having become the seat of morbid changes, there is always a liability to recurrence of the attack from a slighter exciting cause than that which gave rise to the first attack. Hence it is probable that catching cold, in a very large majority of cases, developes in an attack of acute inflammation of some portion of the upper air passages, as being the point of least resistance; and further, as these attacks recur with increased frequency and gravity, we find that the morbid process localizes itself further down and nearer to the vital centres; and finally, this liability so called to take cold, which at first manifests itself in attacks of simple coryza or sore throat, gives rise to a bronchitis or some still graver affection, which fixing itself upon the lungs, may prove far less amenable to treatment than the simpler attacks which preceded it, or even lead to the developement of those still graver forms of pulmonary disease in the management of which our present therapeutic resources are so feeble.

[TO BE CONCLUDED IN NEXT ISSUE.]

